# Indirect genetic control of migration in a salmonid fish

**DOI:** 10.1098/rsbl.2020.0299

**Published:** 2020-08-19

**Authors:** Suzanne J. Kelson, Stephanie M. Carlson, Michael R. Miller

**Affiliations:** 1 Global Water Center, Biology Department, University of Nevada, Reno, NV, USA; 2 Environmental Science, Policy, and Management, University of California, Berkeley, CA, USA; 3 Department of Animal Science, University of California, Davis, CA, USA

**Keywords:** life history, partial migration, *Oncorhynchus mykiss*, steelhead/rainbow trout, genomics, growth

## Abstract

Migration is a complex trait that often has genetic underpinnings. However, it is unclear if migratory behaviour itself is inherited (direct genetic control), or if the decision to migrate is instead the outcome of a set of physiological traits (indirect genetic control). For steelhead/rainbow trout (*Oncorhynchus mykiss*), migration is strongly linked to a large genomic region across their range. Here, we demonstrate a shared allelic basis between early life growth rate and migratory behaviour. Next, we demonstrate that early life growth differs among resident/migratory genotypes in wild juveniles several months prior to migration, with resident genotypes achieving a larger size in their first few months of life than migratory genotypes. We suggest that the genetic basis of migration is likely indirect and mediated by physiological traits such as growth rate. Evolutionary benefits of this indirect genetic mechanism likely include flexibility among individuals and persistence of life-history diversity within and among populations.

## 1. Introduction

Migration influences the distribution of animals across the landscape and their ability to access resources [1]. Many populations include both migratory and non-migratory individuals [2]. For these ‘partially migratory' populations, variation in migratory behaviour often has strong genetic underpinnings across many taxa, from birds to fish [3–5]. However, it is unclear if the behaviour itself is inherited (i.e. direct genetic control), or if the decision to migrate is the outcome of inherited, non-behavioural, physiological traits (i.e. indirect genetic control) [6,7].

There are likely evolutionary benefits to indirect genetic control of migratory behaviour [8], such as phenotypic plasticity, which is common in partially migratory taxa [9–11]. Indirect genetic control via physiological traits could shape the reaction norm for migration within populations [6] thereby influencing the threshold at which individuals express each behaviour [12,13]. The model of indirect genetic control allows for previously observed density-dependent strategies within populations [14] and may help maintain life-history diversity in a population.

The celebrated migrations of salmonid fish, between marine feeding and freshwater breeding grounds, have led to the detailed study of the genetic architecture of migration in these fish. In steelhead/rainbow trout (*Oncorhynchus mykiss*), migration is associated with a large genomic region (*Omy05*) across a broad geographic area [15–18]. *Omy05* has two chromosomal inversions, which leads to reduced recombination, so there could be co-adapted alleles that have been conserved together on this region [19]. It is possible to identify if physiological traits map to this region, and a shared allelic basis between these traits and the region that is known to be associated with migration would reveal one mechanism of indirect genetic control.

Early life growth rate has a strong genetic basis [20,21] and is linked to migration in *O. mykiss* [22–24]. Here, we use the term ‘early life growth' to include development rate (time to hatch) and early life growth rate because they are correlated and map to the same genetic position [25]. Early life growth rate is an important trait because salmonids face strong selection on emergence timing and size [26]. Additionally, given the importance of dominance hierarchies in drift-feeding salmonid fishes [27,28], any competitive edge that is obtained in the first few months may have downstream fitness and life-history consequences [29–31].

The effects of growth rate on migratory behaviour are context-dependent in *O. mykiss* [22]. In some cases, rapid growth is associated with freshwater maturation [32–35], but in others, rapid growth is associated with ocean migration [36–38]. In short, growth alone is an unreliable predictor of migration across streams with different thermal regimes and prey resources. Consequently, there is no clear prediction about whether migratory or resident genotypes are more likely to be associated with rapid early life growth.

Given the relevancy of growth for migratory behaviour, we predicted that the genetic basis for the two is linked. We used published sequence data to reveal a shared allelic basis between the two. This finding prompted us to ask if this shared allelic basis influences growth in wild populations, where growth is a result of many factors. We addressed this question via a field study, comparing size-at-age for greater than 1500 juvenile migratory and resident genotype *O. mykiss*. We use this combination of genetic and ecological data to explore if the genetic basis of migration operates indirectly through physiological traits such as growth.

## 2. Methods

### (a) Shared allelic basis for growth and migration

We explored the overlap in alleles for migration and early life growth. Two clonal lines, Clearwater and Swanson, have faster early life growth in a laboratory than a third line, Whale Rock [20,21]. Previous analyses revealed that the rapid growth of Clearwater and Swanson is controlled by a large, conserved haplotype on *Omy05* [20], the same region associated with migration [15–17]. However, the extent of shared allelic variation between early life growth and migration has not been analysed. Using published sequence data [17], we identified SNPs that are located between 25 and 80 Mb on *Omy05*, the double chromosomal inversion associated with migration [39] and that differ in the major allele between our previously identified resident and migratory genotype groups [16]. These SNPs have low minor allele frequencies within each group (mean ± s.d. of 0.12 ± 0.10 in resident and 0.13 ± 0.11 in migratory genotypes [40]). We then used published sequence data [41] to identify which of these SNPs have also been genotyped in the clonal lines. We identified a set of 128 di-allelic SNPs for which we compared alleles between the three clonal lines and the major allele for the resident and migratory genotype groups. SNPs are available at [40]. We visualized the raw genetic distance between all groups using a neighbour-joining tree produced with the ‘ape' package [42] in *R* [43].

### (b) Field comparison of growth in wild fish

We captured *O. mykiss* from two tributaries to the South Fork Eel River, California, USA, with co-occurring migratory and resident fish: Fox Creek (2.7 km^2^ drainage area) and Elder Creek (16.8 km^2^). Elder Creek has two fish-bearing tributaries, Paralyze (4.9 km^2^) and Misery (1.9 km^2^). We consider Fox Creek and three regions of Elder Creek (above and below a waterfall that is a partial barrier to migration and Paralyze) separately because they differ in migratory/resident genotype frequencies [17]. We excluded Misery because we captured only one migratory genotype out of 64 juveniles.

We captured fish from study pools that encompassed the entire fish-bearing length of the streams from late July to early August each summer from 2014 to 2017 following methods in [17]. Sampling occurred over as few days as possible (less than 5 days per sample location and within three weeks overall) to reduce growth during the sampling window (dates and data are available in [44]). Briefly, we used three-pass electrofishing to estimate fish density in each sample pool, which strongly influences growth rates of juvenile salmonids [45,46]. We captured 2400 juveniles (less than 1 year of age, fork length ≤ 85 mm [47]). We focus on the fork length-at-summer's end for these fish (body size), which represents growth rates over the first few months of life. Early life growth is relevant because it influences whether or not fish reach a threshold for migration [35,36].

We extracted DNA from caudal fin samples and performed restriction site-associated DNA (RAD) capture following [48]. We used SNPs on *Omy05* to assign individuals to resident, heterozygous or migratory genotypes, details in [16], resulting in 1903 genotyped juveniles. We previously demonstrated that these genotypes are associated with migratory behaviour later in life in these streams [16]. Sex can also inform migratory behaviour [16,49], but we expect an even sex ratio among juveniles. In fact, the ratio of males-to-females is not statistically different from 1 : 1 for the subset of juveniles for which we have both sex and migratory/resident genotype data [40] (*n* = 195 juveniles, *p* > 0.4 for binomial tests comparing sex ratios within each genotype).

We compared body size among genotypes with linear mixed-effects models (normal distribution) using R packages ‘lme4' [50] and ‘lmerTest' [51]. First, we compared body size among genotypes within each sample location. Body size is the response variable, genotype is a fixed effect, and location and year are random effects to account for repeated sampling. Next, we compared body size for each genotype across fish densities. We included body size as the response variable, genotype and fish density (individual m^−2^) as fixed effects, a genotype × density interaction, and sample pool (unique for each year) as a random effect. Finally, we compared condition factor among genotypes (discussed in electronic supplementary material).

## 3. Results

### (a) Shared allelic basis for growth and migration

**Figure 1.**
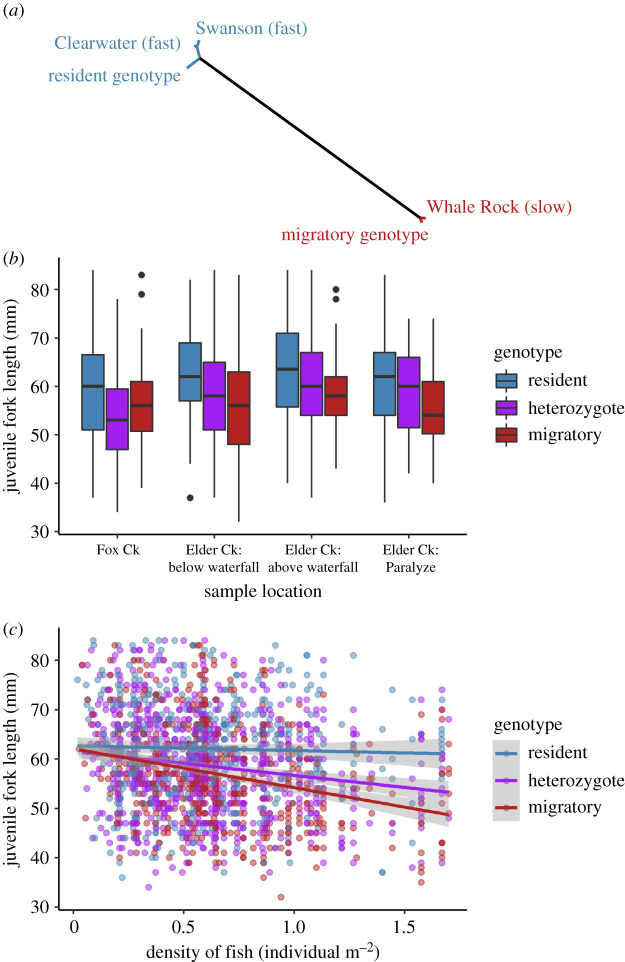
Indirect genetic control of migration in *O. mykiss*. (*a*) Unrooted tree (raw genetic distance) shows clustering among the resident genotypes and faster growing clonal lines and between the migratory genotypes and the slower growing clonal line. Resident genotype juveniles are larger than migratory genotype juveniles several months (at least) before migration across sample locations (*b*) and fish densities (*c*). In (*c*), each point is an individual, lines represent predicted slopes and intercepts, and shading represents one standard error.

We found that in the *Omy05* region, alleles for clonal lines with rapid early life growth (Swanson and Clearwater) were shared with the major allele from resident genotypes at 90.6% and 88.3% of SNPs, but only at 3.1% of SNPs for the slow-growing clonal line (Whale Rock). An unrooted tree demonstrates clustering of the fast-growing lines with the resident genotype and the slow-growing line with the migratory genotype (figure 1*a*). This analysis demonstrates a shared allelic basis between rapid early life growth and residency.

### (b) Field comparison of growth in wild fish

Resident genotype juveniles tended to be slightly larger than migratory genotype juveniles within each location (figure 1*b*). Summarizing across locations, the fork length (mm, mean ± s.d.) for resident, heterozygous and migratory genotypes are 62.1 ± 10.2, 58.4 ± 9.9 and 56.7 ± 9.8, respectively. Genotype is significant in the linear mixed-effects model comparing size within sample locations and years (*F*_2,1743_ = 30.6, *p* < 0.001, electronic supplementary material), with resident genotype fish being larger than migratory genotype fish (contrast from migratory genotype, estimate (Est) ± standard error (s.e.): 5.0 ± 0.7, *p* < 0.001), and heterozygous genotype fish expressing an intermediate size (Est ± s.e.: 1.5 ± 0.6, *p* < 0.01).

Similarly, we found that resident genotype juveniles tended to be larger than migratory genotypes under high fish densities (figure 1*c*). This result indicates that within pools, where rearing conditions are shared, resident genotype juveniles obtain a larger size than migratory genotypes. Size decreased with fish density (*F*_1,196_ = 23.0, *p* < 0.001, Est ± s.e.: −7.3 ± 1.2, *p* < 0.001, electronic supplementary material), but there was a significant interaction effect between density and genotype (*F*_2,1729_ = 7.2, *p* < 0.001). The slope estimate for resident genotype fish was shallower than that for migratory genotype fish, with a contrast of 6.7 ± 1.8 (*p* < 0.001), and heterozygous genotype fish again showing an intermediary level, with a contrast of 2.8 ± 1.4 (*p* = 0.05). This result suggests that at higher densities, resident genotype juveniles tend to be larger than migratory genotype juveniles. We note that the size distributions for fish of each genotype overlap considerably (figure 1*b*,*c*), and similarly that the total explained variance in our models is low (electronic supplementary material). This overlap in body size is expected for fish that are co-rearing, and any observed size differences in these wild, uncontrolled conditions are remarkable.

## 4. Discussion

**Figure 2.**
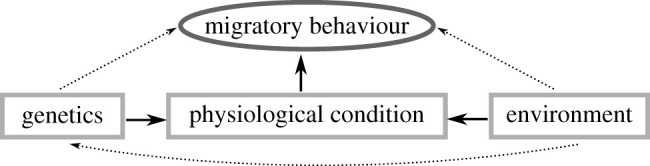
Genetic and environmental effects directly influence physiological condition, which in turn directly influences migratory behaviour. Environment may also influence genetics via epigenetic regulation of gene expression. Dashed arrows represent possible effects that have yet to be observed.

We demonstrate a shared allelic basis between early life growth and migratory behaviour and that this genetic basis is correlated with early life growth in wild fish. This work builds on previous comparisons of growth and life history by comparing growth among *genotypes* rather than phenotypes. We compare growth several months prior to the expression of migration, rather than a post-hoc comparison. This combination of genetic association and field studies allows us to demonstrate that genetic variation influences growth rates far in advance of migration and deepens our understanding of the underlying mechanisms of migration. Our results suggest a model of indirect genetic control of migratory behaviour, such that genetic variation, along with environmental and epigenetic factors, influences physiology, which then influences migratory behaviour (figure 2).

Benefits of indirect genetic control of migration might include flexibility to take advantage of environmental conditions to express the most beneficial life-history strategy. For example, with indirect genetic control, during poor growth conditions, a higher proportion of individuals than expected from genetics may migrate. Indirect genetic control is consistent with many observations of life-history flexibility in wild salmonids. For example, flooding and increased food availability resulted in elevated rates of freshwater maturation (residency) in *Salmo salar* [52]. Similarly, *O. mykiss* isolated in a reservoir for 70 years are still able to express migration [53]. In summary, indirect genetic control may facilitate opportunistic responses to environmental conditions and also encourage the persistence of life-history diversity, contributing to population stability [54].

We found that rapid early life growth is linked to the resident genotype for *O. mykiss*. Mixed results have been reported in other studies comparing growth rate between migratory and resident *O. mykiss*. For example, *O. mykiss* from populations that experience high temperatures and faster growth were more likely to smolt (prepare for migration) compared to slower growing populations [55]. In another study, fast growth was associated with freshwater maturation [35]. These differences may be related to compensatory growth, wherein migratory individuals grow faster depending on the time window and proximity to the outmigration period [56]. We emphasize that growth trajectories are context dependent, and future studies should incorporate how *Omy05* genotypes respond to varying temperature and food conditions.

Beyond differences in growth rate, another factor contributing to variation in size of juvenile *O. mykiss* could be differences in breeding time and the resultant juvenile emergence time. However, for *O. mykiss*, resident fish typically breed a few weeks later than anadromous fish, with some overlap [57–59]. This pattern leads to the expectation that resident fish would emerge from the gravel *later* than their migratory counterparts. However, despite their potential younger age, resident genotype fish were *larger* than migratory genotype fish, providing further support for faster growth rates. There may also be microhabitat differences in nest sites between resident and migratory adults that influence juvenile growth [59], but we compare fish within habitat units.

An alternative model is that *Omy05* directly influences migration behaviour and early life growth differences are a result of previously determined migration decisions. However, the influence of *Omy05* on growth begins very early in life, during embryonic development [20,21,25]. Furthermore, other studies suggest that conditions later in life influence life-history decisions, reviewed in [22]. Second, growth is independent of migration in clonal lines reared in controlled conditions. The Swanson and Clearwater lines have the rapid growth haplotype, but the Clearwater and slower growing Whale Rock lines undergo smoltification [41]. The fact that *Omy05* genotypes are not necessarily correlated with smoltification in clonal lines provides further evidence that this genomic region influences physiological traits, which then influence migratory decisions. These lines of evidence suggest that the decision to migrate or not is context dependent, and informed by physiological condition, which is to some extent genetically controlled.

Other topics that warrant further investigation are the role of epigenetics and sex, relative to indirect genetic mechanisms, in determining migratory behaviour. Epigenetics may be especially important within partially migratory populations where genetic variation is low and environmental conditions are shared. In the Clearwater line, smoltification is associated with several DNA methylated regions [60], suggesting that epigenetics may facilitate behavioural plasticity within genotypes. Epigenetic differences are correlated with migratory behaviours in other taxa as well [61,62] but the relative role of epigenetics in shaping plasticity is unknown [63]. Furthermore, sex can also have a strong influence on migratory decisions in partially migratory populations [2,64], including in our streams [16]. Sexes may differ in their size thresholds needed to initiate migration [33] especially when fitness benefits of migration vary greatly between the sexes [39]. We suggest that future studies investigate how sexes differ in physiological traits and decision thresholds within life-history genotypes and epigenetic modifications.

In summary, we highlight that the genetic basis for migration in *O. mykiss* is likely indirect and mediated by physiological traits. Evolutionary benefits of this indirect genetic mechanism include individual flexibility and persistence of life-history variation. Growth is associated with life-history strategies across many taxa [2], and *O. mykiss* provide a template for how the genetic basis of the two is linked. Studies on the genetic mechanisms of migration illuminate the relative role of genetics in maintaining life-history diversity.

## Supplementary Material

Electronic Supplementary Material

## Data accessibility

Sequence data are available at NCBI, SRA accession: PRJNA599015 and in [20,41]. Fish size and density data are available on Dryad [44]: https://doi.org/10.6078/D1DM6G. Information on SNPs used for allelic comparison and juvenile sex data are available on Dryad [40]: https://doi.org/10.6078/D1TM5T.
