# Supplementary material for: Indirect genetic control of migration in a salmonid fish
Source: Biol Lett. 2020 Aug 19;16(8):20200299. doi: 10.1098/rsbl.2020.0299 (PMC7480158; doi:10.1098/rsbl.2020.0299)
Supplement: Electronic Supplementary Material [file rsbl20200299supp1.docx]

**Electronic Supplementary Material for “Indirect genetic control of migration in a salmonid fish,” Kelson, Suzanne J.^1*^, Carlson, Stephanie M.^2^, Miller, Michael R.^3^**

^1^Global Water Center, Biology Department, University of Nevada, Reno

^2^Environmental Science, Policy, and Management, University of California, Berkeley

^3^ Department of Animal Science, University of California, Davis

*Corresponding author: [skelson@unr.edu](mailto:skelson@unr.edu)

**Supplementary Tables**

**Table 1.** Sample sizes for juveniles in each genotype group, used in Figure 1B and statistical model described in Methods, and summarized in Table 2.

| **Location** | **Migratory Genotype** | **Heterozygote Genotype** | **Resident Genotype** |
| --- | --- | --- | --- |
| Fox Creek | 88 | 79 | 51 |
| Elder Creek: Below waterfall | 380 | 471 | 130 |
| Elder Creek: Above waterfall | 74 | 171 | 192 |
| Elder Creek: Paralyze | 22 | 58 | 123 |

**Table 2.** Parameter estimates for model estimating body size from genotype (fixed effect), nested within year and location (random effects). Restricted maximum likelihood is 13654.9 and log likelihood is -6,827.4. χ^2^ = 61.2, P < 0.001 for a likelihood ratio test comparing this model with a model that does not include genotype as a fixed effect, which has a log likelihood of -6,858.1. Conditional r^2^ for the full model is 0.07 and marginal r^2^ is 0.04, calculated with ‘MuMIN’ package in R (Barton 2019).

| **Random Effects:** | **Variance** | **Std. Dev.** |  |  |  |
| --- | --- | --- | --- | --- | --- |
| Year | 0.6 | 0.8 |  |  |  |
| Location | 3.1 | 1.8 |  |  |  |
| Residual | 97.8 | 9.9 |  |  |  |
|  |  |  |  |  |  |
| **Fixed Effects:** | **Estimate** | **Std. Error** | **Degrees of Freedom** | **t-value** | **P-value** |
| Intercept | 56.8 | 1.0 | 4.8 | 52.7 | <0.001 |
| Genotype – Heterozygote | 1.5 | 0.6 | 1835.9 | 2.7 | <0.01 |
| Genotype – Resident | 5.0 | 0.7 | 1667.5 | 7.7 | <0.001 |

**Table 3.** Parameter estimates for model estimating body size from genotype and fish density (fixed effects), nested within each study pool, unique for every year (random effect). Restricted maximum likelihood is 13,311.4 and log likelihood is -6,655.7. χ^2^ = 86.9, P < 0.001 for a likelihood ratio test comparing this model with a model that does not include genotype as a fixed effect, which has a log likelihood of -6,699.2. Conditional r^2^ for the full model is 0.17 and marginal r^2^ is 0.08, calculated with ‘MuMIN’ package in R (Barton 2019).

| **Random Effects:** | **Variance** | **Std. Dev.** |  |  |  |
| --- | --- | --- | --- | --- | --- |
| Study Pool | 10.0 | 3.2 |  |  |  |
| Residual | 87.8 | 9.4 |  |  |  |
|  |  |  |  |  |  |
| **Fixed Effects:** | **Estimate** | **Std. Error** | **Degrees of Freedom** | **t-value** | **P-value** |
| Intercept | 61.7 | 0.9 | 789.2 | 66.2 | <0.001 |
| Fish/m^2^ | -7.2 | 1.2 | 518.1 | -5.9 | <0.001 |
| Genotype – Heterozygote | -0.3 | 1.1 | 1792.9 | -0.3 | 0.74 |
| Genotype – Resident | 1.0 | 1.3 | 1510.7 | 0.7 | 0.46 |
| Fish/m^2^ × Genotype –Heterozygote | 2.8 | 1.4 | 1765.8 | 1.9 | 0.05 |
| Fish/m^2^ × Genotype –Resident | 6.7 | 1.8 | 1490.8 | 3.8 | <0.001 |

**References**

Barton, K. 2019 MuMIn: Multi-Model Inference. R package version 1.43.10. https://CRAN.R-project.org/package=MuMIn

**Supplementary Analysis on Condition Factor**

We compared the condition factor of migratory and resident genotype juvenile fish by modeling weight (log scale) as a function of body length (log scale) and genotype (García-Berthou 2001). Here, significant main effects of genotype indicate that genotypes differ in their mass given a certain body length. We found slight differences in body condition for migratory and resident genotype juveniles. The main effects for genotype were significant (F_2_ = 4.7, P < 0.01). Here, the resident genotype juveniles were slightly lower in body condition, with an estimate of -0.03 ± 0.12 (t=0.3, P = 0.8) in contrast from migratory genotype juveniles, and so were heterozygote juveniles, with an estimate of -0.01 ± 0.11 (t=0.2, P = 0.9). These differences were very slight, but confirms that there may be a trade-off between body size and body condition for juvenile trout, with resident genotype fish obtaining a larger body size but slightly lower condition in early life.

**References**

Garcia-Berthou, E. 2001 On the misuse of residuals in ecology: testing regression residual vs the analysis of covariance. *J Anim.Ecol.* **70**, 708-711.
